# Maladaptive personality traits (DSM-5 AMPD, Criterion B) and depression among medical students in Egypt: a multicentric cross-sectional study

**DOI:** 10.1186/s40359-025-02784-z

**Published:** 2025-05-07

**Authors:** Khaled Elalfy, Ahmed Farrag, Aya Suleiman, Nour Edin Darwish, Yousef Elqady, Mohamed Esmail, Aya Abdelwahed, Huda Wagih, Personality Consortium, Mey E. Bastawi, Omnia S. Elgendy, Zeyad M. Yassin, Hossam A. Ramadan, Ali K. Kaoud, Thomas E. Gergis, Ahmed M. Abbas, Jana M. Farouk, Mohamed M. Eldabaa, Mohamed K. Rifai, Abdelrhman W. Kotb, Ahmed S. Saleh, Hagar A. Ramadan, Huda M. Farag, Abdelrahman A. Elsaba, Aws F. Yousef, Marwa Gallab

**Affiliations:** 1 Faculty of Medicine, Kafrelsheikh University, Kafr El-Sheikh, Egypt; 2 Faculty of Medicine, Mansoura University, Mansoura, Egypt; 3 Department of Medical Parasitology, Faculty of Medicine, Kafrelsheikh University, Kafr El-Shaikh, Egypt

**Keywords:** Maladaptive personality traits, Personality pathology, DSM-5 AMPD, DepRession, Medical students, Egypt, PHQ-9, PID-5-BF

## Abstract

**Background:**

The relationship between maladaptive personality traits and other mental disorders, such as depression, has been underexplored, especially in medical students. Moreover, the prevalence of depression among medical students is greater than that among the general population, increasing their susceptibility to associated psychopathologies. Consequently, this study aims to investigate the relationship between depression and maladaptive personality trait domains on the basis of Criterion B of the DSM-5 Alternative Model for Personality Disorders (DSM-5 AMPD) among medical students while also highlighting relevant sociodemographic factors.

**Methodology:**

A cross-sectional study was conducted from May to September 2024, with participants surveyed through an online questionnaire. The questionnaire included three sections: sociodemographic characteristics, assessment of depression via the Patient Health Questionnaire-9 (PHQ-9), and maladaptive personality traits via the Personality Inventory for DSM-5—Brief Form (PID-5-BF). Statistical analysis was conducted in R via various packages for data cleaning, analysis, and visualization, employing descriptive statistics, regression models, correlation analysis, and reliability tests.

**Results:**

A total of 2,203 students participated in this study, with a marginal female dominance of 1,230 (55.8%). The mean (SD) overall maladaptive trait score was 1.11 (0.54), and that for the PHQ-9 was 11.7 (6.0). Statistical analysis revealed that higher depression scores were more strongly associated with females than with males, whereas maladaptive trait scores revealed no significant sex differences. The linear regression model for maladaptive trait domains revealed a significant association between total PHQ-9 scores and overall personality trait scores (B = 0.05 [0.05, 0.06]; β = 0.61 [0.58, 0.64]; *p* < 0.001). Similarly, another regression model confirmed this association, with overall personality trait scores being statistically significant (B = 7.0 [6.6, 7.3]; β = 0.62 [0.59, 0.65]; *p* < 0.001)".

**Conclusion:**

Our findings revealed a significant correlation between maladaptive personality traits and depression in medical students. Moreover, the strong correlation between depression and negative affect suggests that negative affect may be closely linked to depressive symptoms. Further research is needed to understand the relationship between maladaptive trait domains and depression and how that relationship affects vulnerable groups such as medical students.

**Supplementary Information:**

The online version contains supplementary material available at 10.1186/s40359-025-02784-z.

## Background

Personality disorders (PDs) pose significant challenges in the field of mental health and are characterized by complex presentations and difficulties in both diagnosis and treatment [1]. These challenges can result in inadequate interventions, potentially leading to poor health outcomes for affected patients. Research suggests a link between PDs and a shortened lifespan, highlighting the substantial individual and societal burdens associated with these conditions [2].

To better assess personality disorders, the American Psychiatric Association developed multiple categorizing and diagnostic models. Earlier frameworks for understanding personality pathology, such as the DSM-IV, employed a categorical system for diagnosing personality disorders (PDs) [3]. However, this model has faced considerable criticism due to high comorbidity rates, diagnostic overlap, variability within categories, and arbitrary diagnostic thresholds [4, 5].

To address the limitations of the categorical model, the DSM-5 introduces the Alternative Model for Personality Disorders (AMPD) in Section III while retaining the traditional 10-category PD classification in Section II [6, 7]. The AMPD marks a shift from a strictly categorical approach to a dimensional framework, aiming for a more nuanced assessment of personality pathology [8]. The Alternative Model for Personality Disorders (AMPD) evaluates personality disorders using two fundamental criteria. Criterion A assesses personality functioning, including intrapersonal aspects (identity and self-direction) and interpersonal aspects (empathy and intimacy). Criterion B identifies maladaptive personality traits across five core domains: negative affectivity, detachment, antagonism, disinhibition, and psychoticism, as defined by the Personality Inventory for DSM-5–Brief Form (PID-5-BF) [9]. Research suggests that the AMPD’s Pathological Trait Model (Criterion B) aligns well with normal personality traits and may serve as a valuable framework for understanding and organizing mental disorders [10].

Personality disorders frequently coexist with depression and are strongly correlated. Individuals with personality disorders often face long-term emotional instability, relationship challenges, and ineffective coping strategies, increasing their susceptibility to depressive episodes [11]. For instance, a study has identified a strong link between borderline personality disorder (BPD) and depression [12]. Additionally, depression commonly co-occurs with avoidant, dependent, and obsessive–compulsive personality disorders, as classified in the traditional categorical model [13]. The presence of PDs also worsens the severity and prognosis of depression. Patients with both conditions tend to have poorer treatment outcomes compared to those with depression alone, underscoring the importance of early identification and targeted interventions [14]. Furthermore, research has linked depression to specific personality traits, such as neuroticism, a key component of the Five-Factor Model (FFM) [15], and emotional dysregulation [16].

Medical students represent a high-risk population for psychological distress, including depression [17]. Research indicates that more than a quarter of medical students globally report symptoms of major depressive disorder (MDD) during training [18]. The prevalence of depression among medical students is significantly greater than that among the general population and age-matched peers [19, 20]. The transition from a student to a physician is marked by high academic pressure, demanding workloads, competitive environments, limited guidance, and fear of failure, all of which contribute to psychological distress [21, 22]. Consequently, medical students experience high rates of depression and anxiety, further emphasizing the need to assess personality traits as potential risk factors for mental health deterioration.

The literature on maladaptive personality traits in relation to depression is still limited. However, a study involving university students by Sleep et al. found that high scores on a depression scale were linked to detachment, negative affect, psychoticism, and disinhibition. Among these, only detachment and negative affect showed a significant association with internalizing symptoms [23]. Another study reported a connection between negative affect and detachment and depression-related cognitive risk factors [24]. Moreover, two distinct personality profiles have been proposed in relation to mood disorders. One is linked to major depressive disorder (MDD), characterized by negative affectivity, detachment, and disinhibition, and the other is associated with bipolar disorder II (BD-II), characterized by antagonism and psychoticism. These findings suggest that personality traits play a critical role in understanding mood disorders and their underlying temperaments [25].

Despite the widespread use of the PID-5 worldwide and substantial research supporting its validity, only a few studies have been published thus far that use the PID-5 in the Middle East and the Arab world [26–29], with even fewer investigations of its relationship with depression. Given the high prevalence of both personality disorders and depression among medical students [18, 30], this study aimed to explore the possibility of a correlation between depression and maladaptive personality traits and measure the levels of maladaptive personality trait domains on the basis of Criterion B of the DSM-5 alternative model for personality disorders (DSM-5 AMPD) and major depression assessed by the PHQ-9 among medical students in Egypt while also analyzing relevant sociodemographic factors. By addressing this research gap, this study seeks to provide valuable insights that could inform early screening, intervention, and mental health support programs for medical students.

## Methods

### Study design and participants

This is a descriptive cross-sectional study with an analytical component. The study was conducted from May to September 2024, totaling approximately 4 months, and was carried out in multiple phases. During May–June, we focused on the study design, questionnaire development, and ethical approval, and data collection took place between late June and late July 2024. The August–September period was dedicated to data processing, statistical analysis, and manuscript preparation. The inclusion criterion was current medical students in Egyptian universities. Students who had already obtained their medical degree or who did not provide consent were excluded. Participants were recruited from multiple Egyptian universities through online platforms and student groups, ensuring diverse representations of medical students across different demographics.

### Sample size

The sample size was calculated via the samplingbook package [31] in R [32]. The scale used in the study consists of 5 domains, each with a range of 0–3, and a total score ranging from 0–3. To estimate the standard deviation, the range (3) was divided by 4, adhering to the range rule of thumb, resulting in a standard deviation of 0.75. Considering a 95% confidence level, a 0.1 margin of error, and a standard deviation of 0.75, a sample size of 217 was calculated. Assuming a design effect of 10, the final sample size was 2170. A total of 2,203 students participated in the study.

### Data collection

The participants were recruited via a convenience sampling technique through communication channels and social media platforms. Collaborators across Egypt aided in data collection. The survey was administered online via Google Forms. To ensure data security, all the responses were anonymized, and participation was voluntary. To minimize duplicate submissions, we implemented settings that restricted survey responses to one per email and distributed the survey link exclusively through official university platforms and verified medical student groups to ensure that only eligible participants could access and complete the survey. The online survey was distributed via Egyptian college platforms, where the data collection phase lasted from 27 June to 27 July 2024.

### Measurement tools

We used a self-administered online questionnaire that included 3 sections. These questionnaires were administered in their English versions, with no translation performed. The first section collected sociodemographic data, including sex, academic year, university, place of residence, income level, academic performance and previous history of psychiatric diseases. To assess academic performance, students reported their GPA, which was categorized into three groups: 3.0–4.0, 2.0–3.0, and below 2.0. In Egypt, GPA is typically on a 0–4.0 scale, with higher scores indicating better performance.

In the second section, the Patient Health Questionnaire-9 (PHQ-9) scale was used to screen for depression [33]. The PHQ-9 is a 9-item instrument given to patients to screen for the presence and severity of depression. The items are scored on a 0–3 Likert-type scale, with a total score ranging from 0 to 27, with higher scores representing more severe degrees of depression. Numerous studies have confirmed the validity and reliability of the PHQ-9 scale as a valid measure of depression severity both in primary clinical care and in the general public setting [34, 35]. Additionally, the reliability of the PHQ-9 was assessed using Cronbach’s alpha, yielding a coefficient of 0.854, indicating medium internal consistency (see Supplementary File for details).

The final section of the questionnaire included the Personality Inventory for DSM-5—Brief Form (PID-5-BF)—Adult to assess personality disorders [36]. The PID-5-BF is a 25-item self-assessment tool designed for adults aged 18 years and above. It evaluates five personality trait domains, namely, negative affect, detachment, antagonism, disinhibition, and psychoticism, each represented by five items. Responses are scored on a 0–3 Likert scale, with higher scores in any domain reflecting the degree of presence of maladaptive personality traits. The average scores for each domain and for the overall measures are represented on a 4-point scale. Numerous studies have thoroughly investigated the reliability and validity of the PID-5 in Egyptian and Middle Eastern samples, consistently demonstrating its reliability as a measurement tool. These studies have consistently reported high internal consistency coefficients, indicating that the PID-5 reliably measures the intended constructs [37, 38]. Several studies have also investigated the correlation between the full-length PID-5 and the brief version, and the resulting data support the use of the PID-5-BF as a screening measure of dimensional maladaptive personality traits [39, 40]. Additionally, the reliability of the PID-5-BF was assessed using Cronbach’s alpha, yielding a coefficient of 0.905, indicating high internal consistency (see Supplementary File for details). Moreover, the PID-5-BF has been deemed valid in numerous other regions, including Europe [41], North America [42], and Asia [43]. In addition, several studies have applied the PID-5-BF for screening among university students and clinical samples [42, 43].

### Data analysis

Statistical analysis was performed via the R programming language and RStudio [32]. Data cleaning, analysis, and table generation were conducted via packages such as tidyverse [44], gtsummary [45], and sjPlot [46]. Categorical variables are summarized as frequencies and percentages, whereas continuous variables are presented as the means and standard deviations. To identify the factors associated with personality traits and depression, both univariate and multivariate linear regression models were employed. Additionally, a correlation matrix was constructed to examine the relationships between the PHQ-9 and PID-5-BF scores via Pearson correlation coefficients. Linear regression analysis was also conducted for the PID-5-BF domains. The results of this analysis are provided in the supplementary file. Additionally, descriptive statistics and reliability analyses were performed for both the PHQ-9 and the PID-5-BF. Detailed findings from these analyses are also included in the supplementary file for further reference. We assessed multicollinearity in our regression models using the generalized variance inflation factor (GVIF), and all GVIF^(1/(2*df)) values were below 2, indicating no concern for multicollinearity. A *p* value less than 0.05 was considered statistically significant.

## Results

### Descriptive statistics

A total of 2,203 students participated in the study, with 1,230 females (55.8%) and 973 males (44.2%). The largest group was Grade 4 (540 participants, 24.5%), followed by Grade 2 (442 participants, 20.1%), and the smallest groups were Grade 1 (238 participants, 10.8%) and Grade 6 (41 participants, 1.86%). The intern group included 310 participants (14.1%). Most participants lived in urban areas (1,277, 58.0%), with 926 (42.0%) living in rural areas. With respect to academic performance, 74.4% had GPAs of 3.0 or higher, 23.0% had GPAs between 2.0 and 3.0, and 2.63% had GPAs below 2.0. Most students lived with their families (1,250, 56.7%), followed by those in dormitories (649, 29.5%) and those living alone (304, 13.8%). In terms of income, 48.5% reported “just enough income,” 30.5% reported “sufficient or exceeded” income, and 21.1% reported “not sufficient” income. Most participants (83.1%) had no history of psychiatric diseases, whereas 16.9% reported such a history (Table 1).

**Table 1:** Descriptive statistics of study variables

**Variable**	***N* = 2,203**^a^
**Sex**	
Female	1,230 (55.83%)
Male	973 (44.17%)
**Grade**	
1	238 (10.80%)
2	442 (20.06%)
3	301 (13.66%)
4	540 (24.51%)
5	331 (15.02%)
6	41 (1.86%)
Intern	310 (14.07%)
**Residence**	
Urban	1,277 (57.97%)
Rural	926 (42.03%)
**GPA**	
< 2	58 (2.63%)
2–3	507 (23.01%)
3–4	1,638 (74.35%)
**Housing**	
Home with family	1,250 (56.74%)
College dormitory	649 (29.46%)
Home alone	304 (13.80%)
**Income**	
Enough and exceeds	671 (30.46%)
Enough only	1,068 (48.48%)
Not enough	464 (21.06%)
**Past history of psychiatric disease**	
No	1,831 (83.11%)
Yes	372 (16.89%)

^a^n (%)

The mean (SD) depression score according to the PHQ-9 was 11.7 (6.0), and that of the overall maladaptive trait domain interpreted by the PID-5-BF was 1.11 (0.54) (Table 2). With respect to individual maladaptive domains, negative affect had the highest score of 1.34 (0.72), followed by detachment at 1.16 (0.67), psychoticism at 1.13 (0.68), disinhibition at 1.02 (0.66) and finally antagonism at 0.91 (0.64) (Table 2).

**Table 2:** Descriptive statistics of PHQ-9 and PID-5-BF Scores

Characteristic	Range	Mean (SD)
PHQ-9	0–27	11.73 (6.02)
Overall Personality Dysfunction	0–3	1.11 (0.54)
Negative Affect	0–3	1.34 (0.72)
Detachment	0–3	1.16 (0.67)
Antagonism	0–3	0.91 (0.64)
Disinhibition	0–3	1.02 (0.66)
Psychoticism	0–3	1.13 (0.68)

### Associated factors of overall maladaptive trait scores

Multivariate regression analysis revealed several statistically significant variables associated with the overall maladaptive trait scores. Males presented higher scores for overall maladaptive traits (B = 0.05 [0.02, 0.09]; β = 0.10 [0.03, 0.16]; *p* = 0.003). Similarly, students from rural areas presented significantly higher scores than did those from urban areas (B = 0.05 [0.02, 0.09]; β = 0.10 [0.03, 0.16]; *p* = 0.004). Moreover, compared with students with a GPA of 3–4, those with a GPA of 2–3 had significantly higher maladaptive trait scores (B = 0.05 [0.01, 0.10]; β = 0.10 [0.02, 0.18]; *p* = 0.011), whereas those with a GPA < 2 presented the highest trait scores (B = 0.13 [0.02, 0.24]; β = 0.25 [0.04, 0.45]; *p* = 0.017). Furthermore, living in a college dormitory had significantly more scores (B = 0.09 [0.05, 0.13]; β = 0.16 [0.09, 0.24]; *p* < 0.001). Finally, participants with a positive psychiatric history demonstrated significantly higher scores (B = 0.17 [0.12, 0.22]; β = 0.32 [0.23, 0.40]; *p* < 0.001) (Table 3).

**Table 3:** Linear regression analysis of overall personality dysfunction scores

**Variable**	**Overall Personality Dysfunction**^*a*^	**Univariate regression**			**Multivariate regression**		
		**Unstandardized Beta (95% CI)**^*b*^	**Standardized Beta (95% CI)**^*b*^	***p*-value**	**Unstandardized Beta (95% CI)**^*b*^	**Standardized Beta (95% CI)**^*b*^	***p*-value**
**Sex**							
Female	1.11 (0.51)	—	—		—	—	
Male	1.11 (0.56)	0.00 (−0.04, 0.05)	0.00 (−0.08, 0.09)	0.934	0.05 (0.02, 0.09)	0.10 (0.03, 0.16)	**0.003**
**Grade**							
1	1.15 (0.54)	—	—		—	—	
2	1.13 (0.54)	−0.02 (−0.11, 0.06)	−0.04 (−0.20, 0.11)	0.595	−0.01 (−0.08, 0.05)	−0.02 (−0.14, 0.10)	0.688
3	1.17 (0.49)	0.02 (−0.07, 0.11)	0.03 (−0.14, 0.20)	0.693	0.00 (−0.07, 0.07)	0.00 (−0.13, 0.13)	0.983
4	1.08 (0.53)	−0.07 (−0.15, 0.01)	−0.13 (−0.28, 0.03)	0.104	−0.04 (−0.11, 0.02)	−0.08 (−0.20, 0.04)	0.173
5	1.07 (0.57)	−0.08 (−0.17, 0.01)	−0.14 (−0.31, 0.02)	0.093	−0.05 (−0.12, 0.02)	−0.09 (−0.22, 0.04)	0.170
6	1.27 (0.69)	0.12 (−0.06, 0.29)	0.22 (−0.12, 0.55)	0.202	0.10 (−0.04, 0.23)	0.18 (−0.07, 0.43)	0.164
Intern	1.08 (0.53)	−0.07 (−0.16, 0.02)	−0.13 (−0.30, 0.04)	0.134	0.02 (−0.05, 0.09)	0.04 (−0.09, 0.17)	0.576
**Residence**							
Urban	1.10 (0.52)	—	—		—	—	
Rural	1.13 (0.56)	0.03 (−0.02, 0.07)	0.05 (−0.04, 0.13)	0.280	0.05 (0.02, 0.09)	0.10 (0.03, 0.16)	**0.004**
**GPA**							
3–4	1.07 (0.53)	—	—		—	—	
2–3	1.22 (0.54)	0.15 (0.10, 0.21)	0.28 (0.19, 0.38)	** < 0.001**	0.05 (0.01, 0.10)	0.10 (0.02, 0.18)	**0.011**
< 2	1.31 (0.57)	0.24 (0.10, 0.38)	0.44 (0.18, 0.70)	** < 0.001**	0.13 (0.02, 0.24)	0.25 (0.04, 0.45)	**0.017**
**Housing**							
Home with family	1.06 (0.54)	—	—		—	—	
College dormitory	1.18 (0.54)	0.12 (0.07, 0.17)	0.22 (0.12, 0.31)	** < 0.001**	0.09 (0.05, 0.13)	0.16 (0.09, 0.24)	** < 0.001**
Home alone	1.18 (0.51)	0.12 (0.05, 0.18)	0.22 (0.09, 0.34)	** < 0.001**	0.05 (−0.01, 0.10)	0.08 (−0.01, 0.18)	0.091
**Income**							
Enough only	1.13 (0.50)	—	—		—	—	
Not enough	1.17 (0.56)	0.04 (−0.02, 0.09)	0.07 (−0.04, 0.18)	0.224	−0.02 (−0.06, 0.03)	−0.03 (−0.11, 0.06)	0.505
Enough and exceeds	1.04 (0.56)	−0.10 (−0.15, −0.05)	−0.18 (−0.28, −0.08)	** < 0.001**	−0.03 (−0.07, 0.00)	−0.06 (−0.14, 0.01)	0.085
**Past history of psychiatric disease**							
No	1.05 (0.52)	—	—		—	—	
Yes	1.41 (0.54)	0.35 (0.30, 0.41)	0.66 (0.55, 0.77)	** < 0.001**	0.17 (0.12, 0.22)	0.32 (0.23, 0.40)	** < 0.001**
**PHQ-9**		0.06 (0.05, 0.06)	0.64 (0.60, 0.67)	** < 0.001**	0.05 (0.05, 0.06)	0.61 (0.58, 0.64)	** < 0.001**

^*a*^Overall personality dysfunction score: Mean (SD)

^*b*^*CI* Confidence Interval

### Factors associated with depression

Multivariate regression analysis revealed several statistically significant variables associated with PHQ-9 scores. Males had lower total PHQ-9 scores (B = −1.2 [−1.6, −0.78]; β = −0.20 [−0.26, −0.13]; *p* < 0.001). Similarly, interns had lower scores than Grade 1 interns did (B = −0.96 [−1.8, −0.17]; β = −0.16 [−0.29, −0.03]; *p* = 0.018). Similarly, residents in rural areas scored lower on the total PHQ-9 scale (B = −0.90 [−1.3, −0.51]; β = −0.15 [−0.21, −0.08]; *p* < 0.001). Moreover, income level had a statistically significant effect on scores. Specifically, the group reporting “enough and exceeds” income had lower scores (B = −0.53 [−0.98, −0.08]; β = −0.09 [−0.16, −0.01]; *p* = 0.021), whereas those who reported “not enough” income had higher scores (B = 0.70 [0.18, 1.02]; β = 0.12 [0.03, 0.20]; *p* = 0.008). Finally, participants who reported a “positive past psychiatric history” had significantly higher scores (B = 0.82 [0.28, 1.4]; β = 0.14 [0.05, 0.22]; *p* = 0.003) (Table 4).

**Table 4:** Linear regression analysis of PHQ-9 scores

**Variable**	**PHQ-9**^*a*^		**Univariate regression**						**Multivariate regression**				
			**Unstandardized Beta (95% CI)**^*2*^		**Standardized Beta (95% CI)**^*2*^		***p*-value**		**Unstandardized Beta (95% CI)**^*2*^		**Standardized Beta (95% CI)**^*b*^		***p*-value**
**Sex**													
Female	12.25 (5.84)		—		—				—		—		
Male	11.06 (6.18)		−1.2 (−1.7, −0.69)		−0.20 (−0.28, −0.11)		** < 0.001**		−1.2 (−1.6, −0.78)		−0.20 (−0.26, −0.13)		** < 0.001**
**Grade**													
1	11.69 (6.35)		—		—				—		—		
2	12.09 (6.03)		0.39 (−0.56, 1.3)		0.07 (−0.09, 0.22)		0.417		0.39 (−0.34, 1.1)		0.06 (−0.06, 0.18)		0.298
3	12.33 (6.04)		0.64 (−0.38, 1.7)		0.11 (−0.06, 0.28)		0.221		0.30 (−0.49, 1.1)		0.05 (−0.08, 0.18)		0.458
4	11.79 (5.86)		0.10 (−0.82, 1.0)		0.02 (−0.14, 0.17)		0.832		0.19 (−0.51, 0.90)		0.03 (−0.09, 0.15)		0.589
5	11.46 (6.13)		−0.23 (−1.2, 0.77)		−0.04 (−0.20, 0.13)		0.651		0.11 (−0.66, 0.88)		0.02 (−0.11, 0.15)		0.784
6	11.80 (6.49)		0.11 (−1.9, 2.1)		0.02 (−0.31, 0.35)		0.913		−0.54 (−2.1, 0.98)		−0.09 (−0.34, 0.16)		0.485
Intern	10.83 (5.79)		−0.87 (−1.9, 0.15)		−0.14 (−0.31, 0.02)		0.094		−0.96 (−1.8, −0.17)		−0.16 (−0.29, −0.03)		**0.018**
**Residence**													
Urban		12.06 (5.88)		—		—				—		—	
Rural		11.27 (6.19)		−0.79 (−1.3, −0.28)		−0.13 (−0.22, −0.05)		**0.002**		−0.90 (−1.3, −0.51)		−0.15 (−0.21, −0.08)	** < 0.001**
**GPA**													
3–4		11.43 (6.06)		—		—				—		—	
2–3		12.63 (5.81)		1.2 (0.61, 1.8)		0.20 (0.10, 0.30)		** < 0.001**		0.18 (−0.30, 0.65)		0.03 (−0.05, 0.11)	0.466
< 2		12.43 (5.97)		1.0 (−0.57, 2.6)		0.17 (−0.09, 0.43)		0.210		−0.63 (−1.8, 0.59)		−0.10 (−0.31, 0.10)	0.312
**Housing**													
Home with family		11.54 (6.06)		—		—				—		—	
College dormitory		11.93 (6.15)		0.39 (−0.18, 0.97)		0.07 (−0.03, 0.16)		0.176		−0.38 (−0.82, 0.06)		−0.06 (−0.14, 0.01)	0.093
Home alone		12.07 (5.58)		0.53 (−0.22, 1.3)		0.09 (−0.04, 0.21)		0.166		−0.34 (−0.94, 0.25)		−0.06 (−0.16, 0.04)	0.255
**Income**													
Enough only		11.92 (5.74)		—		—				—		—	
Not enough		12.48 (6.20)		0.56 (−0.10, 1.2)		0.09 (−0.02, 0.20)		0.094		0.70 (0.18, 1.2)		0.12 (0.03, 0.20)	**0.008**
Enough and exceeds		10.90 (6.26)		−1.0 (−1.6, −0.44)		−0.17 (−0.27, −0.07)		** < 0.001**		−0.53 (−0.98, −0.08)		−0.09 (−0.16, −0.01)	**0.021**
**Past history of psychiatric disease**													
No		11.17 (5.88)		—		—				—		—	
Yes		14.48 (5.98)		3.3 (2.7, 4.0)		0.55 (0.44, 0.66)		** < 0.001**		0.82 (0.28, 1.4)		0.14 (0.05, 0.22)	**0.003**
**Overall personality dysfunction score**				7.1 (6.8, 7.5)		0.64 (0.60, 0.67)		** < 0.001**		7.0 (6.6, 7.3)		0.62 (0.59, 0.65)	** < 0.001**

^*a*^PHQ-9 score: Mean (SD)

^*b*^CI = Confidence Interval

### PID-5-BF and PHQ-9 association

The linear regression model for the overall maladaptive trait domains revealed statistically significant association between total PHQ-9 scores and overall personality trait scores (B = 0.05 [0.05, 0.06]; β = 0.61 [0.58, 0.64]; *p* < 0.001) (Table 3).

Furthermore, in the other linear regression model examining associations of the total PHQ-9 score, the overall personality trait score was statistically significant (B = 7.0 [6.6, 7.3]; β = 0.62 [0.59, 0.65]; *p* < 0.001) (Table 4).

All correlation coefficients were positive and statistically significant (*P* < 0.001). A moderate positive correlation was found between the PHQ-9 score and the overall maladaptive personality trait domain score (*r* = 0.63). Among the domains, negative affectivity had the strongest correlation with depression (*r* = 0.58), followed by psychoticism (*r* = 0.54), detachment (*r* = 0.53), disinhibition (*r* = 0.51), and antagonism, which had the weakest correlation (*r* = 0.31) (Table 5).

**Table 5:** Correlation matrix of PHQ-9 and personality traits

	PHQ-9	Overall Personality Dysfunction	Negative Affect	Detachment	Antagonism	Disinhibition	Psychoticism
PHQ-9							
Overall Personality Dysfunction	0.64^***^ [.61,.66]						
Negative Affect	0.59^***^ [.56,.62]	0.78^***^ [.77,.80]					
Detachment	0.53^***^ [.50,.56]	0.80^***^ [.79,.82]	0.55^***^ [.52,.58]				
Antagonism	0.32^***^ [.28,.35]	0.72^***^ [.70,.74]	0.39^***^ [.36,.43]	0.48^***^ [.44,.51]			
Disinhibition	0.52^***^ [.49,.55]	0.82^***^ [.80,.83]	0.56^***^ [.53,.58]	0.56^***^ [.53,.58]	0.51^***^ [.48,.54]		
Psychoticism	0.55^***^ [.52,.58]	0.84^***^ [.83,.85]	0.58^***^ [.55,.61]	0.60^***^ [.58,.63]	0.52^***^ [.49,.55]	0.62^***^ [.59,.65]	

Pearson Correlation

^***^p < 0.001

Values in square brackets indicate the 95% confidence interval for each correlation

## Discussion

This study aimed to investigate the relationships between depression and maladaptive personality trait domains among Egyptian medical students while also analyzing possible associated factors with sociodemographic characteristics.

Various studies have highlighted the associations between personality traits and other comorbidities [47]. According to the PHQ-9 depression results, the PID-5-BF results, and the Pearson correlation coefficients, there was a strong positive correlation between higher levels of depression and overall personality trait scores. Specifically, depression showed a statistically significant correlation with negative affect, detachment, disinhibition, and psychoticism, with negative affect exhibiting the strongest correlation. Moreover, a Middle Eastern study on Iranian samples examining the correlation between maladaptive personality traits and depression found that patients with major depressive disorder had significantly higher scores in negative affectivity, detachment, and disinhibition compared to healthy controls [25]. In addition, negative affect had the highest association, suggesting that the key to this similarity between personality traits and depression could be related primarily to the correlation between negative affect and depression, which has been highlighted in multiple studies [48, 49]. Antagonism was the only maladaptive facet that showed a weak correlation with depression, which lowers the possibility of it being associated with depression compared to other traits [25, 47, 49].

A study using PID-5 normative data [50] from Krueger et al. [51] reported lower scores across all maladaptive trait domains compared to our findings. Our results were closer to those of their clinical samples, suggesting more severe maladaptive trait expression in medical students. However, the lack of national normative data for the PHQ-9 and PID-5-BF in Egypt limits contextualization, and cultural differences may affect comparability with international norms.

With respect to sociodemographic characteristics, the overall maladaptive personality trait scores favored males. Moreover, all the maladaptive domains except for detachment, exhibited sex differences with negative affect being more associated with females while antagonism, disinhibition and psychoticism were more related to males. In line with these results, Suzuki et al. performed a study measuring sex invariance of the DSM-5 Section III pathological personality trait model which found that females tend to have higher scores on latent negative affectivity, whereas males tend to have higher scores on latent antagonism, detachment, psychoticism, and disinhibition [52]. These consistent findings further support the idea that men are generally expected to score higher on the PID-5 Trait domains, with the exception of negative affectivity domain [52, 53]. Low academic performance was also associated with higher maladaptive trait scores. This is consistent with previous literature arguing that personality traits have a robust association with performance along with emotional stability and academic achievements [54]. Moreover, having a history of psychiatric illnesses showed significant association with higher maladaptive trait scores, which is supported by literature highlighting the comorbidity of personality pathology and higher occurrence with different illnesses [11, 14]. Other characteristics, such as rural residence and living in college dormitories, were associated with significantly higher maladaptive scores. However, research on the relation between residence and maladaptive traits is scarce, while findings from studies on rural–urban differences in Big Five traits found no significant differences in personality changes [55]. Further research is needed to explore how residence influences maladaptive traits and how they compare to normal personality traits.

Depression was expressed more in females than in males, which is consistent with other Egyptian studies on medical students reporting the same findings [56, 57]. These results can be explained by females being more likely to report stress, high load of curriculum, physical and psychological symptoms [56]. In addition, the academic year of medical students was also significantly associated with depression, with students in their intern years having lower depressive symptom scores. This is consistent with previous literature highlighting that the prevalence of depression was more associated with students in their first three years, while also reporting a decrease in depressive symptoms with the advance of academic years [57]. A possible explanation to this pattern is the gradual adaptation of students to the new academic environment and getting accustomed to the stress of learning and meeting academic responsibilities [17]. Low-income levels were associated with higher levels of depression, which is supported by similar Egyptian and foreign studies [57, 58]. This can be explained by various factors including chronic stress that comes with financial instability, limited access to resources and other social determinants [57, 59]. Having a history of psychiatric illnesses was also significant with higher depression scores. To complement these results, Egyptian studies reporting on the depressive symptoms of medical students in both upper Egypt and Alexandria reported that a history of mental illness was strongly associated with increased depressive symptoms [17, 48]. Finally, living in rural areas was associated with lower depressive symptom scores, which is supported by other literature measuring depressive symptoms in medical students [48, 57, 58].

This study provides invaluable insights into the degree of correlation between maladaptive traits and depression while providing data on significant demographic correlations with both of these mental illnesses among Egyptian medical students. By identifying the specific trait domains most strongly associated with depression and their sociodemographic patterns, this study enhances our understanding of mental health and underscores the need for early detection and intervention strategies such as awareness campaigns and early mental health screening programs, which could play crucial roles in supporting vulnerable students.

However, this study has several limitations. The cross-sectional study design does not address how maladaptive traits change over time and cannot establish causal relationships. A potential way to mitigate these issues is to perform longitudinal observational studies. In addition, convenience sampling makes the data gathered prone to selection bias, and the self-reported nature of the survey makes this study liable to information bias. A possible way to alleviate these limitations is through the use of a different sampling technique, such as random sampling, to provide accurate results that can be effectively generalized. Moreover, since the data were collected from anonymized participants, ensuring the lack of duplicates or nonmedical student submissions was not possible. Subsequent research could improve participant verification by implementing additional eligibility checks or email authentication. Further research is suggested to address these limitations.

## Conclusion

Maladaptive personality traits and depression are significantly positively correlated in medical students. More specifically, negative affect had the strongest association with depression out of all the maladaptive traits. Most of the other maladaptive traits also had a positive correlation with depression, making medical students and any group in which depression is prevalent very vulnerable to these correlative traits. In the multivariate linear regression model, maladaptive personality trait scores were significantly associated with males, rural residence, low academic performance, living in college dormitories and past history of psychiatric illnesses. Additionally, depression scores were significantly associated with females, year of education, urban residency, low income, and past history of psychiatric illnesses. These findings emphasize the need for tailored mental health interventions and further research, including cross-cultural studies, to better understand and address the psychological challenges faced by medical students. Early detection and support are crucial to improving their academic and personal well-being.

## Supplementary Information

Supplementary Material 1.

## Data Availability

The datasets generated and analyzed during this study are not publicly available due to the lack of participant consent for sharing anonymized data. However, they can be obtained from the corresponding author upon reasonable request.

## Abbreviations

PID-5-BF: Personality Inventory for DSM-5—Brief Form
PHQ-9: Patient Health Questionnaire-9
