# Supplementary material for: Maladaptive personality traits (DSM-5 AMPD, Criterion B) and depression among medical students in Egypt: a multicentric cross-sectional study
Source: BMC Psychol. 2025 May 7;13:482. doi: 10.1186/s40359-025-02784-z (PMC12060542; doi:10.1186/s40359-025-02784-z)
Supplement: Supplementary file 1 — Supplementary Material 1. [file 40359_2025_2784_MOESM1_ESM.docx]

Table 1: Linear regression analysis of Negative Affect scores

| **Variable** | **Negative Affect***^1^* | **Univariate regression** | | | **Multivariate regression** | | |
| --- | --- | --- | --- | --- | --- | --- | --- |
|  |  | **Unstandardized Beta** **(95% CI)***^2^* | **Standardized Beta** **(95% CI)***^2^* | **p-value** | **Unstandardized Beta** **(95% CI)***^2^* | **Standardized Beta** **(95% CI)***^2^* | **p-value** |
| **Sex** |  |  |  |  |  |  |  |
| Female | 1.45 (0.71) | — | — |  | — | — |  |
| Male | 1.20 (0.71) | -0.24 (-0.30, -0.18) | -0.34 (-0.42, -0.26) | **<0.001** | -0.23 (-0.28, -0.19) | -0.32 (-0.38, -0.26) | **<0.001** |
| **Grade** |  |  |  |  |  |  |  |
| 1 | 1.35 (0.76) | — | — |  | — | — |  |
| 2 | 1.35 (0.73) | 0.01 (-0.11, 0.12) | 0.01 (-0.15, 0.17) | 0.908 | -0.04 (-0.12, 0.04) | -0.05 (-0.16, 0.06) | 0.368 |
| 3 | 1.41 (0.71) | 0.07 (-0.05, 0.19) | 0.09 (-0.08, 0.26) | 0.275 | 0.01 (-0.08, 0.09) | 0.01 (-0.11, 0.13) | 0.908 |
| 4 | 1.31 (0.69) | -0.03 (-0.14, 0.08) | -0.04 (-0.20, 0.11) | 0.572 | -0.05 (-0.13, 0.03) | -0.07 (-0.18, 0.04) | 0.225 |
| 5 | 1.31 (0.70) | -0.04 (-0.16, 0.08) | -0.06 (-0.22, 0.11) | 0.503 | -0.02 (-0.11, 0.06) | -0.03 (-0.15, 0.09) | 0.611 |
| 6 | 1.25 (0.76) | -0.09 (-0.33, 0.15) | -0.13 (-0.46, 0.20) | 0.449 | -0.16 (-0.32, 0.01) | -0.22 (-0.45, 0.01) | 0.067 |
| Intern | 1.33 (0.76) | -0.02 (-0.14, 0.10) | -0.03 (-0.20, 0.14) | 0.751 | 0.00 (-0.09, 0.09) | 0.00 (-0.12, 0.12) | >0.999 |
| **Residence** |  |  |  |  |  |  |  |
| Urban | 1.35 (0.71) | — | — |  | — | — |  |
| Rural | 1.32 (0.73) | -0.03 (-0.09, 0.04) | -0.04 (-0.12, 0.05) | 0.410 | 0.00 (-0.04, 0.04) | 0.00 (-0.06, 0.06) | 0.958 |
| **GPA** |  |  |  |  |  |  |  |
| 3-4 | 1.32 (0.71) | — | — |  | — | — |  |
| 2-3 | 1.38 (0.74) | 0.06 (-0.01, 0.13) | 0.08 (-0.02, 0.18) | 0.102 | -0.08 (-0.13, -0.03) | -0.11 (-0.18, -0.04) | **0.002** |
| <2 | 1.43 (0.77) | 0.11 (-0.08, 0.30) | 0.15 (-0.11, 0.41) | 0.257 | -0.08 (-0.22, 0.05) | -0.11 (-0.30, 0.07) | 0.238 |
| **Housing** |  |  |  |  |  |  |  |
| Home with family | 1.31 (0.74) | — | — |  | — | — |  |
| College dormitory | 1.39 (0.69) | 0.07 (0.01, 0.14) | 0.10 (0.01, 0.20) | **0.034** | 0.00 (-0.05, 0.05) | 0.00 (-0.06, 0.07) | 0.926 |
| Home alone | 1.34 (0.73) | 0.02 (-0.07, 0.11) | 0.03 (-0.09, 0.16) | 0.600 | -0.04 (-0.11, 0.02) | -0.06 (-0.15, 0.03) | 0.203 |
| **Income** |  |  |  |  |  |  |  |
| Enough only | 1.39 (0.70) | — | — |  | — | — |  |
| Not enough | 1.38 (0.74) | -0.01 (-0.08, 0.07) | -0.01 (-0.12, 0.10) | 0.885 | -0.03 (-0.09, 0.03) | -0.04 (-0.12, 0.04) | 0.325 |
| Enough and exceeds | 1.23 (0.74) | -0.15 (-0.22, -0.08) | -0.21 (-0.31, -0.11) | **<0.001** | -0.08 (-0.13, -0.03) | -0.11 (-0.18, -0.04) | **0.002** |
| **Past history of psychiatric disease** |  |  |  |  |  |  |  |
| No | 1.27 (0.71) | — | — |  | — | — |  |
| Yes | 1.69 (0.70) | 0.42 (0.34, 0.50) | 0.58 (0.47, 0.69) | **<0.001** | 0.11 (0.05, 0.17) | 0.15 (0.07, 0.23) | **<0.001** |
| **PHQ-9** |  | 0.07 (0.07, 0.07) | 0.59 (0.56, 0.62) | **<0.001** | 0.03 (0.03, 0.04) | 0.26 (0.22, 0.30) | **<0.001** |
| **Detachment** |  | 0.59 (0.55, 0.63) | 0.55 (0.51, 0.58) | **<0.001** | 0.17 (0.13, 0.21) | 0.16 (0.12, 0.20) | **<0.001** |
| **Antagonism** |  | 0.44 (0.40, 0.48) | 0.39 (0.35, 0.43) | **<0.001** | 0.06 (0.01, 0.10) | 0.05 (0.01, 0.09) | **0.008** |
| **Disinhibition** |  | 0.61 (0.57, 0.65) | 0.56 (0.52, 0.59) | **<0.001** | 0.21 (0.17, 0.26) | 0.19 (0.15, 0.23) | **<0.001** |
| **Psychoticism** |  | 0.61 (0.58, 0.65) | 0.58 (0.54, 0.61) | **<0.001** | 0.21 (0.16, 0.25) | 0.19 (0.15, 0.24) | **<0.001** |
| *^1^*Negative Affect score: Mean (SD)  *^2^*CI = Confidence Interval | | | | | | | |

Table 2: Linear regression analysis of Detachment scores

| **Variable** | **Detachment***^1^* | **Univariate regression** | | | **Multivariate regression** | | |
| --- | --- | --- | --- | --- | --- | --- | --- |
|  |  | **Unstandardized Beta** **(95% CI)***^2^* | **Standardized Beta** **(95% CI)***^2^* | **p-value** | **Unstandardized Beta** **(95% CI)***^2^* | **Standardized Beta** **(95% CI)***^2^* | **p-value** |
| **Sex** |  |  |  |  |  |  |  |
| Female | 1.16 (0.66) | — | — |  | — | — |  |
| Male | 1.16 (0.69) | 0.00 (-0.05, 0.06) | 0.01 (-0.08, 0.09) | 0.874 | 0.02 (-0.02, 0.07) | 0.03 (-0.03, 0.10) | 0.289 |
| **Grade** |  |  |  |  |  |  |  |
| 1 | 1.18 (0.70) | — | — |  | — | — |  |
| 2 | 1.17 (0.67) | -0.01 (-0.11, 0.10) | -0.01 (-0.17, 0.15) | 0.923 | 0.02 (-0.06, 0.10) | 0.03 (-0.08, 0.14) | 0.603 |
| 3 | 1.23 (0.65) | 0.05 (-0.07, 0.16) | 0.07 (-0.10, 0.24) | 0.415 | 0.04 (-0.04, 0.12) | 0.06 (-0.06, 0.19) | 0.324 |
| 4 | 1.14 (0.69) | -0.04 (-0.14, 0.06) | -0.06 (-0.21, 0.09) | 0.439 | 0.03 (-0.05, 0.10) | 0.04 (-0.07, 0.15) | 0.459 |
| 5 | 1.12 (0.68) | -0.06 (-0.17, 0.05) | -0.09 (-0.26, 0.08) | 0.290 | 0.02 (-0.06, 0.10) | 0.03 (-0.09, 0.15) | 0.603 |
| 6 | 1.31 (0.72) | 0.13 (-0.09, 0.36) | 0.20 (-0.13, 0.53) | 0.242 | 0.05 (-0.11, 0.21) | 0.08 (-0.16, 0.32) | 0.527 |
| Intern | 1.13 (0.64) | -0.05 (-0.16, 0.07) | -0.07 (-0.24, 0.10) | 0.401 | 0.05 (-0.03, 0.14) | 0.08 (-0.04, 0.21) | 0.205 |
| **Residence** |  |  |  |  |  |  |  |
| Urban | 1.16 (0.67) | — | — |  | — | — |  |
| Rural | 1.16 (0.68) | 0.00 (-0.06, 0.06) | 0.00 (-0.09, 0.08) | 0.962 | -0.01 (-0.05, 0.03) | -0.02 (-0.08, 0.05) | 0.615 |
| **GPA** |  |  |  |  |  |  |  |
| 3-4 | 1.11 (0.66) | — | — |  | — | — |  |
| 2-3 | 1.30 (0.67) | 0.20 (0.13, 0.26) | 0.29 (0.19, 0.39) | **<0.001** | 0.06 (0.01, 0.11) | 0.09 (0.02, 0.17) | **0.015** |
| <2 | 1.41 (0.75) | 0.30 (0.12, 0.47) | 0.44 (0.18, 0.70) | **<0.001** | 0.11 (-0.02, 0.24) | 0.17 (-0.03, 0.36) | 0.091 |
| **Housing** |  |  |  |  |  |  |  |
| Home with family | 1.11 (0.67) | — | — |  | — | — |  |
| College dormitory | 1.22 (0.67) | 0.11 (0.05, 0.17) | 0.16 (0.07, 0.26) | **<0.001** | 0.02 (-0.03, 0.06) | 0.02 (-0.05, 0.09) | 0.499 |
| Home alone | 1.26 (0.68) | 0.15 (0.07, 0.24) | 0.23 (0.10, 0.35) | **<0.001** | 0.04 (-0.02, 0.11) | 0.07 (-0.03, 0.16) | 0.170 |
| **Income** |  |  |  |  |  |  |  |
| Enough only | 1.19 (0.65) | — | — |  | — | — |  |
| Not enough | 1.22 (0.67) | 0.03 (-0.04, 0.10) | 0.05 (-0.06, 0.16) | 0.398 | -0.01 (-0.07, 0.04) | -0.02 (-0.10, 0.06) | 0.609 |
| Enough and exceeds | 1.08 (0.71) | -0.11 (-0.17, -0.04) | -0.16 (-0.26, -0.07) | **<0.001** | -0.02 (-0.07, 0.03) | -0.03 (-0.10, 0.04) | 0.406 |
| **Past history of psychiatric disease** |  |  |  |  |  |  |  |
| No | 1.10 (0.66) | — | — |  | — | — |  |
| Yes | 1.45 (0.68) | 0.34 (0.27, 0.42) | 0.51 (0.40, 0.62) | **<0.001** | 0.02 (-0.04, 0.07) | 0.03 (-0.06, 0.11) | 0.544 |
| **PHQ-9** |  | 0.06 (0.06, 0.06) | 0.53 (0.50, 0.57) | **<0.001** | 0.02 (0.02, 0.02) | 0.18 (0.14, 0.22) | **<0.001** |
| **Negative Affect** |  | 0.51 (0.48, 0.54) | 0.55 (0.51, 0.58) | **<0.001** | 0.16 (0.12, 0.19) | 0.17 (0.12, 0.21) | **<0.001** |
| **Antagonism** |  | 0.50 (0.46, 0.54) | 0.48 (0.44, 0.51) | **<0.001** | 0.16 (0.12, 0.20) | 0.15 (0.11, 0.19) | **<0.001** |
| **Disinhibition** |  | 0.57 (0.53, 0.60) | 0.56 (0.52, 0.59) | **<0.001** | 0.14 (0.10, 0.18) | 0.14 (0.09, 0.18) | **<0.001** |
| **Psychoticism** |  | 0.60 (0.57, 0.63) | 0.60 (0.57, 0.64) | **<0.001** | 0.23 (0.19, 0.28) | 0.24 (0.19, 0.28) | **<0.001** |
| *^1^*Detachment score: Mean (SD)  *^2^*CI = Confidence Interval | | | | | | | |

Table 3: Linear regression analysis of Antagonism scores

| **Variable** | **Antagonism***^1^* | **Univariate regression** | | | **Multivariate regression** | | |
| --- | --- | --- | --- | --- | --- | --- | --- |
|  |  | **Unstandardized Beta** **(95% CI)***^2^* | **Standardized Beta** **(95% CI)***^2^* | **p-value** | **Unstandardized Beta** **(95% CI)***^2^* | **Standardized Beta** **(95% CI)***^2^* | **p-value** |
| **Sex** |  |  |  |  |  |  |  |
| Female | 0.85 (0.62) | — | — |  | — | — |  |
| Male | 0.99 (0.66) | 0.15 (0.10, 0.20) | 0.23 (0.15, 0.32) | **<0.001** | 0.12 (0.07, 0.16) | 0.18 (0.11, 0.25) | **<0.001** |
| **Grade** |  |  |  |  |  |  |  |
| 1 | 0.94 (0.64) | — | — |  | — | — |  |
| 2 | 0.88 (0.63) | -0.06 (-0.16, 0.04) | -0.10 (-0.26, 0.06) | 0.223 | -0.01 (-0.09, 0.07) | -0.01 (-0.14, 0.11) | 0.841 |
| 3 | 0.99 (0.62) | 0.05 (-0.06, 0.16) | 0.07 (-0.10, 0.24) | 0.397 | 0.08 (0.00, 0.17) | 0.13 (0.00, 0.27) | 0.057 |
| 4 | 0.89 (0.64) | -0.05 (-0.15, 0.05) | -0.08 (-0.23, 0.07) | 0.311 | 0.05 (-0.03, 0.13) | 0.08 (-0.04, 0.21) | 0.180 |
| 5 | 0.87 (0.66) | -0.07 (-0.18, 0.04) | -0.11 (-0.27, 0.06) | 0.202 | 0.01 (-0.07, 0.10) | 0.02 (-0.11, 0.15) | 0.763 |
| 6 | 1.22 (0.71) | 0.28 (0.07, 0.49) | 0.44 (0.11, 0.77) | **0.009** | 0.20 (0.03, 0.37) | 0.31 (0.05, 0.58) | **0.020** |
| Intern | 0.88 (0.63) | -0.06 (-0.17, 0.05) | -0.10 (-0.27, 0.07) | 0.257 | 0.03 (-0.06, 0.12) | 0.05 (-0.09, 0.19) | 0.466 |
| **Residence** |  |  |  |  |  |  |  |
| Urban | 0.87 (0.63) | — | — |  | — | — |  |
| Rural | 0.96 (0.65) | 0.09 (0.04, 0.15) | 0.14 (0.06, 0.23) | **<0.001** | 0.06 (0.01, 0.10) | 0.09 (0.02, 0.16) | **0.012** |
| **GPA** |  |  |  |  |  |  |  |
| 3-4 | 0.87 (0.63) | — | — |  | — | — |  |
| 2-3 | 1.03 (0.65) | 0.16 (0.09, 0.22) | 0.24 (0.15, 0.34) | **<0.001** | 0.01 (-0.04, 0.06) | 0.02 (-0.07, 0.10) | 0.715 |
| <2 | 1.09 (0.63) | 0.22 (0.05, 0.38) | 0.34 (0.08, 0.60) | **0.011** | -0.01 (-0.15, 0.13) | -0.02 (-0.23, 0.20) | 0.886 |
| **Housing** |  |  |  |  |  |  |  |
| Home with family | 0.83 (0.63) | — | — |  | — | — |  |
| College dormitory | 1.02 (0.63) | 0.18 (0.12, 0.24) | 0.29 (0.19, 0.38) | **<0.001** | 0.10 (0.05, 0.15) | 0.15 (0.08, 0.23) | **<0.001** |
| Home alone | 1.02 (0.64) | 0.18 (0.11, 0.26) | 0.29 (0.16, 0.41) | **<0.001** | 0.09 (0.02, 0.15) | 0.13 (0.03, 0.24) | **0.011** |
| **Income** |  |  |  |  |  |  |  |
| Enough only | 0.93 (0.62) | — | — |  | — | — |  |
| Not enough | 0.98 (0.66) | 0.05 (-0.02, 0.12) | 0.07 (-0.03, 0.18) | 0.179 | 0.00 (-0.05, 0.06) | 0.01 (-0.08, 0.10) | 0.893 |
| Enough and exceeds | 0.84 (0.65) | -0.09 (-0.15, -0.03) | -0.14 (-0.24, -0.04) | **0.004** | -0.03 (-0.08, 0.02) | -0.04 (-0.12, 0.04) | 0.291 |
| **Past history of psychiatric disease** |  |  |  |  |  |  |  |
| No | 0.86 (0.62) | — | — |  | — | — |  |
| Yes | 1.15 (0.71) | 0.28 (0.21, 0.35) | 0.44 (0.33, 0.55) | **<0.001** | 0.06 (0.00, 0.12) | 0.09 (0.00, 0.19) | 0.052 |
| **PHQ-9** |  | 0.03 (0.03, 0.04) | 0.32 (0.28, 0.36) | **<0.001** | -0.01 (-0.01, 0.00) | -0.08 (-0.13, -0.03) | **<0.001** |
| **Negative Affect** |  | 0.35 (0.31, 0.38) | 0.39 (0.35, 0.43) | **<0.001** | 0.06 (0.01, 0.10) | 0.06 (0.02, 0.11) | **0.008** |
| **Detachment** |  | 0.45 (0.42, 0.49) | 0.48 (0.44, 0.51) | **<0.001** | 0.17 (0.13, 0.22) | 0.18 (0.14, 0.23) | **<0.001** |
| **Disinhibition** |  | 0.49 (0.46, 0.53) | 0.51 (0.47, 0.54) | **<0.001** | 0.23 (0.18, 0.27) | 0.24 (0.19, 0.28) | **<0.001** |
| **Psychoticism** |  | 0.49 (0.46, 0.52) | 0.52 (0.49, 0.56) | **<0.001** | 0.24 (0.19, 0.28) | 0.25 (0.20, 0.30) | **<0.001** |
| *^1^*Antagonism score: Mean (SD)  *^2^*CI = Confidence Interval | | | | | | | |

Table 4: Linear regression analysis of Disinhibition scores

| **Variable** | **Disinhibition***^1^* | **Univariate regression** | | | **Multivariate regression** | | |
| --- | --- | --- | --- | --- | --- | --- | --- |
|  |  | **Unstandardized Beta** **(95% CI)***^2^* | **Standardized Beta** **(95% CI)***^2^* | **p-value** | **Unstandardized Beta** **(95% CI)***^2^* | **Standardized Beta** **(95% CI)***^2^* | **p-value** |
| **Sex** |  |  |  |  |  |  |  |
| Female | 0.99 (0.64) | — | — |  | — | — |  |
| Male | 1.06 (0.69) | 0.06 (0.01, 0.12) | 0.10 (0.01, 0.18) | **0.024** | 0.09 (0.05, 0.13) | 0.13 (0.07, 0.19) | **<0.001** |
| **Grade** |  |  |  |  |  |  |  |
| 1 | 1.04 (0.68) | — | — |  | — | — |  |
| 2 | 1.03 (0.69) | -0.02 (-0.12, 0.09) | -0.03 (-0.18, 0.13) | 0.748 | 0.02 (-0.06, 0.09) | 0.02 (-0.09, 0.14) | 0.664 |
| 3 | 1.03 (0.64) | -0.01 (-0.13, 0.10) | -0.02 (-0.19, 0.15) | 0.821 | -0.02 (-0.10, 0.06) | -0.02 (-0.15, 0.10) | 0.684 |
| 4 | 0.98 (0.63) | -0.07 (-0.17, 0.03) | -0.10 (-0.25, 0.05) | 0.191 | 0.02 (-0.06, 0.09) | 0.03 (-0.08, 0.13) | 0.648 |
| 5 | 1.00 (0.69) | -0.05 (-0.16, 0.06) | -0.07 (-0.24, 0.09) | 0.396 | 0.04 (-0.04, 0.12) | 0.06 (-0.05, 0.18) | 0.293 |
| 6 | 1.22 (0.76) | 0.18 (-0.04, 0.40) | 0.27 (-0.06, 0.61) | 0.105 | 0.09 (-0.07, 0.24) | 0.13 (-0.10, 0.37) | 0.260 |
| Intern | 1.06 (0.65) | 0.01 (-0.10, 0.13) | 0.02 (-0.15, 0.19) | 0.816 | 0.13 (0.05, 0.22) | 0.20 (0.08, 0.33) | **0.001** |
| **Residence** |  |  |  |  |  |  |  |
| Urban | 1.00 (0.65) | — | — |  | — | — |  |
| Rural | 1.05 (0.67) | 0.06 (0.00, 0.11) | 0.08 (0.00, 0.17) | 0.050 | 0.05 (0.01, 0.09) | 0.08 (0.02, 0.14) | **0.014** |
| **GPA** |  |  |  |  |  |  |  |
| 3-4 | 0.97 (0.65) | — | — |  | — | — |  |
| 2-3 | 1.14 (0.66) | 0.17 (0.10, 0.23) | 0.25 (0.15, 0.35) | **<0.001** | 0.04 (-0.01, 0.09) | 0.06 (-0.01, 0.13) | 0.112 |
| <2 | 1.23 (0.72) | 0.26 (0.09, 0.43) | 0.39 (0.13, 0.65) | **0.003** | 0.08 (-0.05, 0.20) | 0.12 (-0.07, 0.30) | 0.219 |
| **Housing** |  |  |  |  |  |  |  |
| Home with family | 0.98 (0.66) | — | — |  | — | — |  |
| College dormitory | 1.07 (0.67) | 0.09 (0.02, 0.15) | 0.13 (0.04, 0.22) | **0.007** | -0.02 (-0.06, 0.03) | -0.02 (-0.09, 0.04) | 0.501 |
| Home alone | 1.07 (0.64) | 0.09 (0.01, 0.17) | 0.13 (0.01, 0.26) | **0.037** | -0.02 (-0.08, 0.04) | -0.03 (-0.12, 0.06) | 0.522 |
| **Income** |  |  |  |  |  |  |  |
| Enough only | 1.02 (0.64) | — | — |  | — | — |  |
| Not enough | 1.10 (0.68) | 0.08 (0.01, 0.15) | 0.12 (0.01, 0.23) | **0.029** | 0.02 (-0.04, 0.07) | 0.03 (-0.05, 0.10) | 0.534 |
| Enough and exceeds | 0.97 (0.68) | -0.05 (-0.11, 0.01) | -0.07 (-0.17, 0.02) | 0.127 | 0.06 (0.01, 0.10) | 0.08 (0.02, 0.15) | **0.016** |
| **Past history of psychiatric disease** |  |  |  |  |  |  |  |
| No | 0.97 (0.64) | — | — |  | — | — |  |
| Yes | 1.28 (0.70) | 0.32 (0.24, 0.39) | 0.48 (0.37, 0.59) | **<0.001** | -0.02 (-0.07, 0.04) | -0.02 (-0.10, 0.06) | 0.585 |
| **PHQ-9** |  | 0.06 (0.05, 0.06) | 0.52 (0.48, 0.55) | **<0.001** | 0.02 (0.01, 0.02) | 0.15 (0.11, 0.19) | **<0.001** |
| **Negative Affect** |  | 0.51 (0.48, 0.54) | 0.56 (0.52, 0.59) | **<0.001** | 0.18 (0.14, 0.22) | 0.19 (0.15, 0.24) | **<0.001** |
| **Detachment** |  | 0.55 (0.51, 0.58) | 0.56 (0.52, 0.59) | **<0.001** | 0.13 (0.09, 0.17) | 0.13 (0.09, 0.17) | **<0.001** |
| **Antagonism** |  | 0.52 (0.49, 0.56) | 0.51 (0.47, 0.54) | **<0.001** | 0.19 (0.15, 0.22) | 0.18 (0.15, 0.22) | **<0.001** |
| **Psychoticism** |  | 0.60 (0.57, 0.64) | 0.62 (0.59, 0.65) | **<0.001** | 0.25 (0.21, 0.29) | 0.26 (0.21, 0.30) | **<0.001** |
| *^1^*Disinhibition score: Mean (SD)  *^2^*CI = Confidence Interval | | | | | | | |

Table 5: Linear regression analysis of Psychoticism scores

| **Variable** | **Psychoticism***^1^* | **Univariate regression** | | | **Multivariate regression** | | |
| --- | --- | --- | --- | --- | --- | --- | --- |
|  |  | **Unstandardized Beta** **(95% CI)***^2^* | **Standardized Beta** **(95% CI)***^2^* | **p-value** | **Unstandardized Beta** **(95% CI)***^2^* | **Standardized Beta** **(95% CI)***^2^* | **p-value** |
| **Sex** |  |  |  |  |  |  |  |
| Female | 1.11 (0.67) | — | — |  | — | — |  |
| Male | 1.15 (0.69) | 0.04 (-0.02, 0.09) | 0.05 (-0.03, 0.14) | 0.209 | 0.04 (0.00, 0.09) | 0.07 (0.01, 0.13) | **0.030** |
| **Grade** |  |  |  |  |  |  |  |
| 1 | 1.24 (0.68) | — | — |  | — | — |  |
| 2 | 1.20 (0.70) | -0.04 (-0.14, 0.07) | -0.05 (-0.21, 0.10) | 0.508 | -0.01 (-0.09, 0.06) | -0.02 (-0.13, 0.09) | 0.702 |
| 3 | 1.18 (0.67) | -0.06 (-0.17, 0.06) | -0.09 (-0.26, 0.08) | 0.320 | -0.09 (-0.17, -0.01) | -0.13 (-0.25, -0.02) | **0.024** |
| 4 | 1.09 (0.67) | -0.15 (-0.25, -0.05) | -0.22 (-0.37, -0.07) | **0.005** | -0.10 (-0.17, -0.03) | -0.14 (-0.25, -0.04) | **0.007** |
| 5 | 1.07 (0.68) | -0.16 (-0.28, -0.05) | -0.24 (-0.41, -0.08) | **0.004** | -0.11 (-0.18, -0.03) | -0.16 (-0.27, -0.05) | **0.006** |
| 6 | 1.31 (0.75) | 0.07 (-0.15, 0.30) | 0.11 (-0.22, 0.44) | 0.516 | -0.05 (-0.20, 0.10) | -0.07 (-0.29, 0.15) | 0.523 |
| Intern | 1.01 (0.65) | -0.23 (-0.34, -0.11) | -0.34 (-0.50, -0.17) | **<0.001** | -0.17 (-0.25, -0.09) | -0.25 (-0.37, -0.14) | **<0.001** |
| **Residence** |  |  |  |  |  |  |  |
| Urban | 1.13 (0.67) | — | — |  | — | — |  |
| Rural | 1.13 (0.69) | 0.00 (-0.05, 0.06) | 0.01 (-0.08, 0.09) | 0.896 | -0.02 (-0.06, 0.02) | -0.03 (-0.08, 0.03) | 0.365 |
| **GPA** |  |  |  |  |  |  |  |
| 3-4 | 1.08 (0.67) | — | — |  | — | — |  |
| 2-3 | 1.26 (0.67) | 0.18 (0.12, 0.25) | 0.27 (0.17, 0.37) | **<0.001** | 0.03 (-0.02, 0.08) | 0.05 (-0.02, 0.11) | 0.191 |
| <2 | 1.39 (0.75) | 0.31 (0.13, 0.48) | 0.45 (0.19, 0.71) | **<0.001** | 0.06 (-0.06, 0.18) | 0.09 (-0.08, 0.27) | 0.305 |
| **Housing** |  |  |  |  |  |  |  |
| Home with family | 1.07 (0.67) | — | — |  | — | — |  |
| College dormitory | 1.20 (0.70) | 0.13 (0.07, 0.19) | 0.19 (0.10, 0.29) | **<0.001** | 0.03 (-0.01, 0.07) | 0.04 (-0.02, 0.11) | 0.192 |
| Home alone | 1.21 (0.65) | 0.14 (0.05, 0.22) | 0.20 (0.08, 0.33) | **0.002** | 0.00 (-0.06, 0.06) | 0.00 (-0.08, 0.09) | 0.967 |
| **Income** |  |  |  |  |  |  |  |
| Enough only | 1.15 (0.66) | — | — |  | — | — |  |
| Not enough | 1.18 (0.69) | 0.03 (-0.05, 0.10) | 0.04 (-0.07, 0.15) | 0.481 | 0.00 (-0.06, 0.05) | -0.01 (-0.08, 0.07) | 0.874 |
| Enough and exceeds | 1.07 (0.70) | -0.08 (-0.15, -0.02) | -0.12 (-0.22, -0.03) | **0.013** | 0.00 (-0.04, 0.05) | 0.00 (-0.06, 0.07) | 0.934 |
| **Past history of psychiatric disease** |  |  |  |  |  |  |  |
| No | 1.06 (0.66) | — | — |  | — | — |  |
| Yes | 1.47 (0.69) | 0.41 (0.33, 0.48) | 0.60 (0.49, 0.71) | **<0.001** | 0.08 (0.03, 0.13) | 0.11 (0.04, 0.19) | **0.004** |
| **PHQ-9** |  | 0.06 (0.06, 0.07) | 0.55 (0.51, 0.58) | **<0.001** | 0.02 (0.01, 0.02) | 0.15 (0.11, 0.19) | **<0.001** |
| **Negative Affect** |  | 0.54 (0.51, 0.58) | 0.58 (0.54, 0.61) | **<0.001** | 0.17 (0.13, 0.20) | 0.18 (0.14, 0.21) | **<0.001** |
| **Detachment** |  | 0.61 (0.58, 0.64) | 0.60 (0.57, 0.64) | **<0.001** | 0.20 (0.17, 0.24) | 0.20 (0.16, 0.24) | **<0.001** |
| **Antagonism** |  | 0.55 (0.52, 0.59) | 0.52 (0.49, 0.56) | **<0.001** | 0.19 (0.15, 0.22) | 0.18 (0.14, 0.21) | **<0.001** |
| **Disinhibition** |  | 0.64 (0.60, 0.67) | 0.62 (0.59, 0.65) | **<0.001** | 0.24 (0.20, 0.28) | 0.23 (0.19, 0.27) | **<0.001** |
| *^1^*Psychoticism score: Mean (SD)  *^2^*CI = Confidence Interval | | | | | | | |

Table 6: Descriptive Statistics of PID-5-BF

| **Items & Subscales** | **N** | **Mean** | **SD** | **Median** | **Min** | **Max** | **Skewness** | **Excess Kurtosis** |
| --- | --- | --- | --- | --- | --- | --- | --- | --- |
| Item 1 | 2203 | 0.83 | 0.93 | 1.00 | 0 | 3 | 0.82 | -0.39 |
| Item 2 | 2203 | 1.12 | 0.89 | 1.00 | 0 | 3 | 0.36 | -0.68 |
| Item 3 | 2203 | 1.18 | 0.96 | 1.00 | 0 | 3 | 0.32 | -0.89 |
| Item 4 | 2203 | 1.25 | 0.99 | 1.00 | 0 | 3 | 0.31 | -0.97 |
| Item 5 | 2203 | 0.83 | 0.93 | 1.00 | 0 | 3 | 0.78 | -0.47 |
| Item 6 | 2203 | 1.13 | 0.97 | 1.00 | 0 | 3 | 0.37 | -0.90 |
| Item 7 | 2203 | 1.14 | 0.94 | 1.00 | 0 | 3 | 0.37 | -0.81 |
| Item 8 | 2203 | 1.55 | 1.04 | 2.00 | 0 | 3 | -0.04 | -1.17 |
| Item 9 | 2203 | 1.54 | 1.04 | 2.00 | 0 | 3 | -0.01 | -1.19 |
| Item 10 | 2203 | 1.33 | 1.08 | 1.00 | 0 | 3 | 0.20 | -1.23 |
| Item 11 | 2203 | 1.02 | 0.93 | 1.00 | 0 | 3 | 0.56 | -0.60 |
| Item 12 | 2203 | 0.73 | 0.89 | 0.00 | 0 | 3 | 0.95 | -0.14 |
| Item 13 | 2203 | 1.19 | 1.04 | 1.00 | 0 | 3 | 0.38 | -1.06 |
| Item 14 | 2203 | 0.97 | 0.97 | 1.00 | 0 | 3 | 0.63 | -0.67 |
| Item 15 | 2203 | 1.24 | 0.98 | 1.00 | 0 | 3 | 0.30 | -0.93 |
| Item 16 | 2203 | 1.31 | 1.00 | 1.00 | 0 | 3 | 0.21 | -1.03 |
| Item 17 | 2203 | 0.68 | 0.92 | 0.00 | 0 | 3 | 1.15 | 0.19 |
| Item 18 | 2203 | 1.09 | 0.95 | 1.00 | 0 | 3 | 0.48 | -0.73 |
| Item 19 | 2203 | 1.04 | 0.95 | 1.00 | 0 | 3 | 0.51 | -0.72 |
| Item 20 | 2203 | 1.01 | 0.95 | 1.00 | 0 | 3 | 0.52 | -0.80 |
| Item 21 | 2203 | 1.23 | 1.02 | 1.00 | 0 | 3 | 0.31 | -1.05 |
| Item 22 | 2203 | 0.78 | 0.89 | 1.00 | 0 | 3 | 0.84 | -0.28 |
| Item 23 | 2203 | 1.45 | 1.03 | 1.00 | 0 | 3 | 0.02 | -1.14 |
| Item 24 | 2203 | 1.10 | 1.01 | 1.00 | 0 | 3 | 0.47 | -0.94 |
| Item 25 | 2203 | 1.05 | 0.98 | 1.00 | 0 | 3 | 0.50 | -0.86 |
| Total Score | 2203 | 1.11 | 0.54 | 1.08 | 0 | 3 | 0.31 | 0.17 |
| Negative Affect | 2203 | 1.34 | 0.72 | 1.40 | 0 | 3 | 0.15 | -0.55 |
| Detachment | 2203 | 1.16 | 0.67 | 1.20 | 0 | 3 | 0.31 | -0.42 |
| Antagonism | 2203 | 0.91 | 0.64 | 0.80 | 0 | 3 | 0.54 | -0.14 |
| Disinhibition | 2203 | 1.02 | 0.66 | 1.00 | 0 | 3 | 0.49 | -0.24 |
| Psychoticism | 2203 | 1.13 | 0.68 | 1.00 | 0 | 3 | 0.31 | -0.48 |

Table 7: Reliability Analysis of PID-5-BF

| **Scale** | **# Items** | **Cronbach's α** | **95% CI Lower** | **95% CI Upper** | **Standardized α** | **Mean Inter-item r** |
| --- | --- | --- | --- | --- | --- | --- |
| Negative Affect | 5 | 0.755 | 0.739 | 0.771 | 0.754 | 0.380 |
| Detachment | 5 | 0.708 | 0.689 | 0.728 | 0.711 | 0.330 |
| Antagonism | 5 | 0.715 | 0.696 | 0.734 | 0.716 | 0.335 |
| Disinhibition | 5 | 0.751 | 0.735 | 0.768 | 0.752 | 0.378 |
| Psychoticism | 5 | 0.732 | 0.714 | 0.749 | 0.730 | 0.351 |
| Overall Scale | 25 | 0.905 | 0.899 | 0.910 | 0.905 | 0.277 |

Table 8: Descriptive Statistics of PHQ-9

| **Items** | **N** | **Mean** | **SD** | **Median** | **Min** | **Max** | **Skewness** | **Excess Kurtosis** |
| --- | --- | --- | --- | --- | --- | --- | --- | --- |
| Item 1 | 2203 | 1.41 | 0.98 | 1.00 | 0 | 3 | 0.18 | -0.96 |
| Item 2 | 2203 | 1.41 | 0.94 | 1.00 | 0 | 3 | 0.17 | -0.86 |
| Item 3 | 2203 | 1.55 | 1.04 | 2.00 | 0 | 3 | -0.03 | -1.17 |
| Item 4 | 2203 | 1.70 | 0.94 | 2.00 | 0 | 3 | -0.11 | -0.95 |
| Item 5 | 2203 | 1.28 | 1.01 | 1.00 | 0 | 3 | 0.26 | -1.04 |
| Item 6 | 2203 | 1.46 | 1.03 | 1.00 | 0 | 3 | 0.09 | -1.13 |
| Item 7 | 2203 | 1.36 | 1.01 | 1.00 | 0 | 3 | 0.21 | -1.03 |
| Item 8 | 2203 | 0.75 | 0.93 | 0.00 | 0 | 3 | 1.00 | -0.10 |
| Item 9 | 2203 | 0.80 | 0.99 | 0.00 | 0 | 3 | 0.93 | -0.36 |
| Total Score | 2203 | 11.73 | 6.02 | 11.00 | 0 | 27 | 0.24 | -0.37 |

Table 9: Reliability Analysis of PHQ-9

| **Scale** | **# Items** | **Cronbach's α** | **95% CI Lower** | **95% CI Upper** | **Standardized α** | **Mean Inter-item r** |
| --- | --- | --- | --- | --- | --- | --- |
| PHQ-9 | 9 | 0.854 | 0.845 | 0.863 | 0.854 | 0.394 |
